# Assessment of Definitions of Sustained Disease Progression in Relapsing-Remitting Multiple Sclerosis

**DOI:** 10.1155/2013/189624

**Published:** 2013-03-10

**Authors:** Brian C. Healy, David Engler, Bonnie Glanz, Alexander Musallam, Tanuja Chitnis

**Affiliations:** ^1^Partners MS Center, Harvard Medical School, Brigham and Women's Hospital, 1 Brookline Place, Brookline, MA 02445, USA; ^2^Biostatistics Center, Massachusetts General Hospital, Boston, MA 02114, USA; ^3^Department of Statistics, Brigham Young University, Provo, UT 84602, USA

## Abstract

Sustained progression on the expanded disability status scale (EDSS) is a common outcome measure of disease progression in clinical studies of MS. Unfortunately, this outcome may not accurately measure long-term and irreversible disease progression. To assess the performance of definitions of sustained progression, patients with relapsing-remitting MS (RRMS) or a clinically isolated syndrome with evidence of lesions on a brain MRI were included in our study. Fifteen definitions of sustained progression using both the EDSS and the functional system (FS) scales were investigated. The impact of both relapses and changes in provider on the probability of maintaining progression was also evaluated. Although the provider scoring the EDSS sometimes changed during followup, the provider had access to previous EDSS scores. Between 15.8% and 42.2% of patients experienced sustained progression based on the definitions using EDSS as the outcome, but nearly 50% of these patients failed to maintain sustained progression for the duration of followup. When FS scales were used, progression was most common on the pyramidal and sensory scales. Unfortunately, progression on specific FS scales failed to be more sensitive to irreversible disability. Relapses or changes in provider did not explain the poor performance of the measures. Short-term changes in the EDSS or FS scores may not be an accurate marker of irreversible change in RRMS.

## 1. Introduction

Measurement of disease progression in patients with multiple sclerosis (MS) is complicated by the occurrence of relapses and the potential for recovery, especially early in the disease course in patients with relapsing-remitting MS (RRMS). The most common way to measure disability in MS patients is the expanded disability status scale (EDSS), which is a 0–10 scale with 0.5 point steps based on eight functional system (FS) scales [1]. These FS scales measure seven separate aspects of the disease using five-to seven-step ordinal scales: pyramidal function, cerebellar function, brainstem function, sensory function, bowel/bladder function, mental function, and visual function. The remaining scale is a measure of the presence of other symptoms. In long-term followup studies, EDSS scores of 4, 6, or 7 are considered milestones in terms of disability, and reaching these milestones is a measure of disease progression, especially since patients rarely improve after such milestones are reached [2, 3]. Since the time to these EDSS scores is often long, a short-term indicator of disease progression early in the disease course is an increase in EDSS score that is sustained over several months. This is called sustained disease progression. Sustained disease progression is required because transient increases in the EDSS may also be due to relapses [4], which reflect disease activity rather than disease progression. However, there is still controversy over an optimized definition of short-term disease progression [5].

The ideal measure of sustained progression would accurately identify patients with true disease progression that would not revert given additional followup. Several definitions of sustained progression for clinical trials and observational studies have been proposed, but a one-point increase sustained for at least three or six months for patients with an EDSS of less than 6 (sometimes a 1.5-point increase for EDSS  =  0) or an increase of 0.5 for patients with an EDSS of 6 or greater is probably the most common [6, 7]. Unfortunately, many patients who fulfill this criterion for sustained progression fail to maintain elevated EDSS scores later in the disease course, demonstrating that this outcome does not correctly identify patients with true irreversible disease worsening [8, 9]. Definitions using alternative EDSS changes or time intervals have also been investigated, but no perfect definition has been developed [10]. One potential difficulty for definitions of sustained progression is the complex relationship between changes on specific FS scales and changes on the EDSS scale, especially in patients with mild disability. Therefore, alternative definitions of sustained progression may more accurately measure irreversible disability accumulation, and these may provide better outcomes for clinical trials and clinical research studies.

In this paper, several definitions of disease progression based on the EDSS are assessed, and additional definitions based on the FS scales are explored. For each outcome, the distribution of time to progression was estimated to determine the pattern of disability accumulation in a group of MS patients. Then, the likelihood of maintaining sustained progression for the remainder of followup was estimated to evaluate the performance of each outcome. Third, the potential impact of relapses and changes in provider on the performance of these definitions was assessed. Finally, the implications of the results for clinical trials focused on sustained progression are discussed.

## 2. Materials and Methods

### 2.1. Subjects

**Figure 1:**
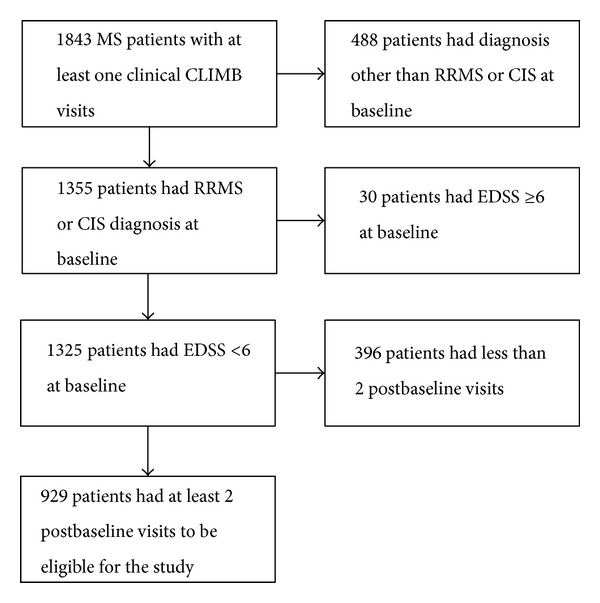
Flow chart for selection of patients included in study.

All patients with RRMS or a clinically isolated syndrome (CIS) with evidence of lesions on a brain MRI enrolled in the comprehensive longitudinal investigation of multiple sclerosis at the Brigham and Women's Hospital and Partners MS Center (CLIMB) as of 3/1/2012 were eligible for our study [11]. Patients were also required to have a baseline EDSS of less than 6 to focus attention on patients likely to be enrolled in clinical trials of RRMS. CLIMB patients have clinical visits every six months. At each clinical visit, a complete neurological exam is completed including measurement of the EDSS and each of the FS scales. The EDSS and FS scales can be scored by different providers at different visits, and the previous EDSS and FS scores are available to the rating provider. For our analysis of sustained progression, patients needed to have at least 1 year of followup. This restriction yielded a final sample of 929 patients. The baseline demographic and clinical characteristics of our sample are provided in Table 1, and a flow chart of patient enrollment is provided in Figure 1. The mean (SD) followup time was 3.7 (2.5) years. This study has received IRB approval from our institution.

### 2.2. Clinical Measures/Definitions of Progression

The clinical outcome measures in our study were the EDSS and FS scores, and definitions of sustained progression were based on these measures. The first four definitions used the EDSS as the outcome, and a specific increase relative to baseline was required at two consecutive visits. These increases were (D1) increase of 0.5 on the EDSS, (D2) increase of 1, (D3) increase of 1.5, and (D4) increase of 1.5 for patients with baseline EDSS  =  0, and increase of 1 for patients with baseline 1 ≤ EDSS ≤ 5.5. Two consecutive visits corresponded to six months for the majority of patients who experienced progression (339/392 for patients who experienced sustained progression using D1). For the remaining subjects, the two visits were separated by 12 (*n* = 40), 18 (*n* = 8), or 24 or more (*n* = 5) months; it was assumed that the EDSS levels observed at these visits were the same as would have been observed at the 6-month visit.

In addition, several definitions based on the FS scores were investigated. First, sustained progression on each FS scale was investigated based on a one-point increase on the scale: (D5) pyramidal, (D6) cerebellar, (D7) brainstem, (D8) sensory, (D9) bowel-bladder, (D10) mental, and (D11) visual. As before, progression was required to be sustained for 2 consecutive visits. The other symptoms' scale was not considered in our analysis. Since sustained progression on the EDSS represents one potential set of changes on the FS scales, four other potential combinations of the FS scores were considered. For the first two, the time to sustained progression was defined as the time to the first sustained progression on any of the FS scales. Then, progression was identified as sustained for the remainder of followup if (D12) any FS scale continued to show progression or (D13) the first FS scale continued to show progression. The final two definitions used the sum of the FS scales and defined sustained progression as an increase of (D14) two points in the sum of the FS scales or (D15) three points in the sum of the FS scales. Although previous work has demonstrated the potential pitfalls of summing the FS scores especially late in the scale [12] and summing the scale is not advocated by the originator of the scale [1], these two definitions were assessed to determine if they were more sensitive to irreversible disease progression than the other definitions in the low end of the EDSS scale where the disadvantages of summing are less clear.

### 2.3. Statistical Analysis

A Kaplan-Meier curve for the time to sustained progression was created for each definition. For definitions based on the EDSS, the FS scales associated with the sustained progression were also determined. In addition to the descriptive characteristics, the effect of baseline EDSS score or FS score on the time to sustained progression was investigated using a Cox proportional hazards model. In each case, the baseline EDSS or FS scores were treated as a continuous predictor, and the model was refit including only patients with EDSS  ≤  3.5 at baseline. 

To investigate the long-term behavior of each measure of sustained progression, each patient was classified based on whether sustained progression was maintained for the rest of followup. The proportion of patients who maintained sustained progression throughout followup was compared across the definitions. In addition, the effect of followup length and baseline functional score on the probability of maintaining sustained progression was investigated using logistic regression. 

Two potential explanations for failing to maintain progression are the presence of relapses and changes in the provider rating the patient. To assess the impact of relapses, all visits classified during a relapse by the treating provider were removed, and sustained progression was investigated using only the nonrelapse visits. The same analyses as above were completed. To assess the impact of changing providers on maintaining sustained progression, the provider at the time of sustained progression was compared to the provider at the time of reversion to determine if a provider change had occurred. As a comparison group, the provider at the time of sustained progression was compared to the provider at last visit for the patients who did not revert. The proportion of patients who changed provider was compared in the two groups using Fisher's exact test to determine if provider changes were associated with reversion.

## 3. Results

### 3.1. Time to Sustained Progression

**Figure 2:**
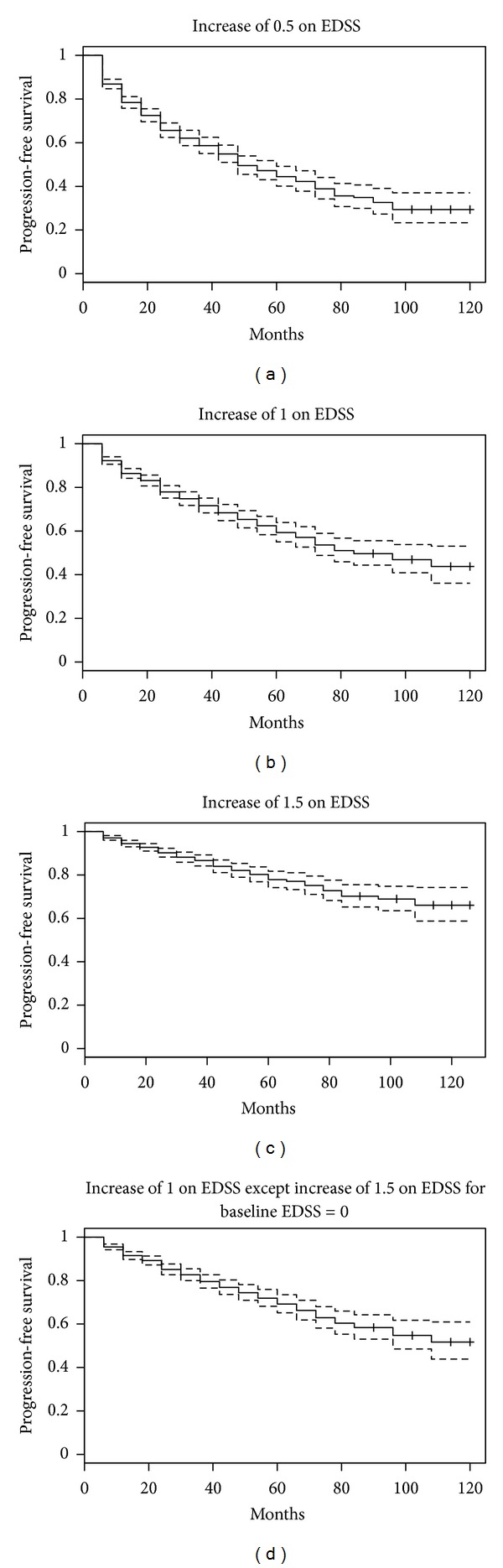
Time to sustained progression for EDSS definitions. EDSS: expanded disability status scale. Each Kaplan-Meier plot presents the time to sustained progression using a specific definition: (a) top left: increase of 0.5 on the EDSS sustained for two visits (D1); (b) top right: increase of 1 on the EDSS sustained for two visits (D2); (c) bottom left: increase of 1.5 on the EDSS sustained for two visits (D3); (d) bottom right: increase of 1.5 for baseline EDSS  =  0 and increase of 1 for baseline EDSS ≥ 1 sustained for two visits (D4).

**Figure 3:**
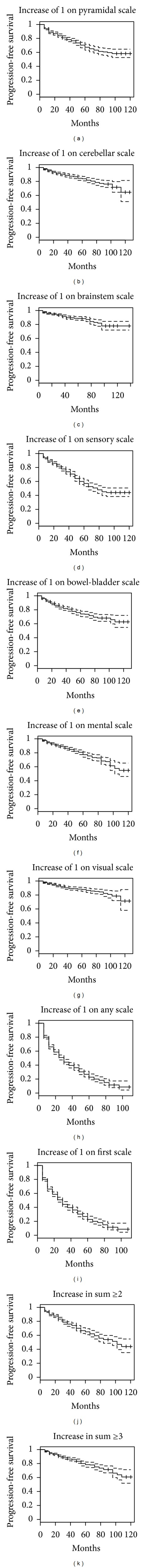
Time to sustained progression for functional system score definitions. Each graph represents a different definition of sustained progression: top row (from left to right)—increase on pyramidal subscale (D5), increase on cerebellar subscale (D6), increase on brainstem subscale (D7), increase on sensory subscale (D8); middle row (from left to right)—increase on bowel-bladder subscale (D9), increase on mental subscale (D10), increase on visual subscale (D11), increase on any subscale (D12); bottom row (from left to right)—increase in first subscale (D13), increase of 2 in the sum of the subscales (D14), increase of 3 in the sum of the subscales (D15).

Using the EDSS-based definitions, between 15.8% and 42.2% of patients were classified as having sustained progression during followup (Table 2), and the time to sustained progression and the corresponding 95% confidence interval are presented in Figure 2. As expected, the time to progression was fastest for D1 and slowest for D3. When sustained progression on each of the individual FS was investigated, the time to sustained progression varied widely across the scales (Figure 3). The time to sustained progression was fastest for the pyramidal and sensory scales and slowest for the brainstem and visual scales. These results demonstrated that progression differentially affects specific FS. Finally, for the alternative definitions of sustained progression based on combinations of the FS scales (D12–D15), the time to sustained progression was fastest for those based on progression on any scale (Figure 3). The time to sustained progression based on a two point increase in the sum of the FS scales was similar to the time for an EDSS increase of 1, and the time to a three-point increase in the sum was similar to the time for an EDSS increase of 1.5. 

Across all EDSS definitions, the most common cause of sustained progression was an increase in sensory or pyramidal symptoms (Table 3). For D1 and D2, between 30% and 41% of patients experienced progression on the pyramidal or sensory scales; while for D3 and D4, between 42% and 57% of patients experienced progression for each of these systems. D1 and D2 were also associated with a greater than 15% chance of progression on no FS scales even though EDSS progression was observed, while progression on no FS scales was much less common for D3 and D4. Finally, over 50% of patients who progressed for D3 and D4 had disease progression on multiple FS scales, demonstrating that the progression was often due to multiple domains.

### 3.2. Effect of Baseline Disability Status on Time to Sustained Progression

**Figure 4:**
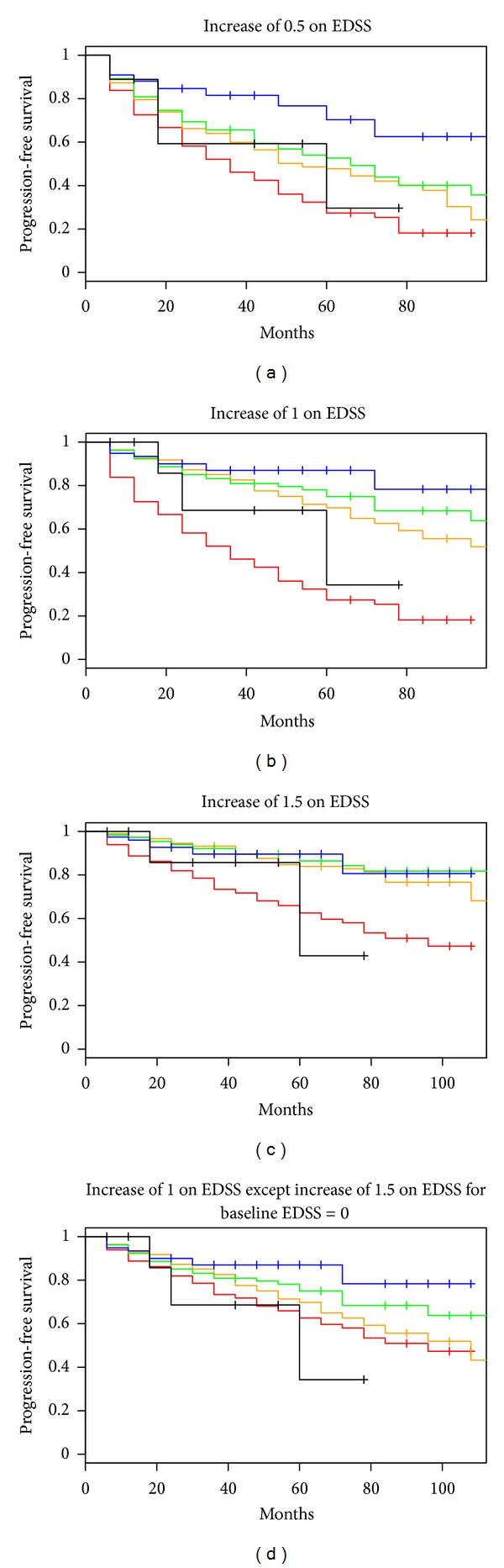
Time to sustained progression stratified by baseline EDSS value. Colors of Kaplan-Meier curve are based on baseline EDSS value: EDSS  =  0: red, EDSS  =  1/1.5: orange, EDSS**   **=  2/2.5: green, EDSS ** **=  3/3.5: blue, EDSS  =  4–5.5: black. Progression definition for each plot: (a) D1, (b) D2, (c) D3, and (d) D4.

Baseline disability level had an important effect on the time to sustained progression (Figure 4). In particular, patients with a baseline EDSS =  0 had a faster time to sustained progression for all of the progression definitions based on changes in the EDSS compared to patients with baseline EDSS between 1 and 3.5. This result was particularly striking when the criterion was D2 (Figure 4(b)). A decreasing hazard of progression with increasing baseline EDSS was observed for all definitions, and the effects were very strong for D1–D3 (*P* < 0.0001) and significant for D4 (*P* = 0.03). These effects were even stronger when baseline EDSS was restricted to be less than 4. When baseline EDSS was less than 4, the hazard of progression decreased by a factor of 0.51 (95% CI: 0.44, 0.59) for each one-point increase in the EDSS for D2 and 0.83 (95% CI: 0.72, 0.96) for D4; the hazard ratios for the other definitions were between these values.

**Figure 5:**
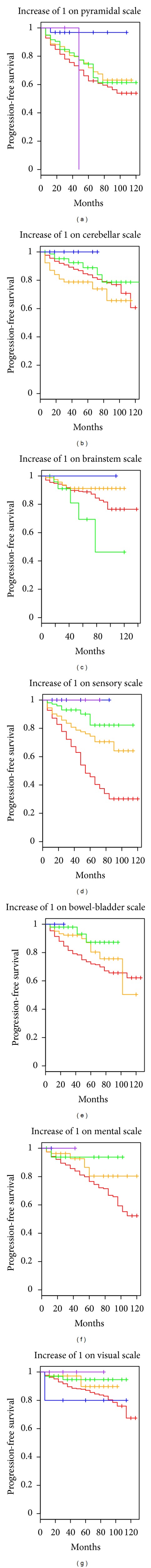
Time to sustained progression stratified by baseline functional system score value. Colors indicate baseline functional system score: score  =  0: red, score  =   1: orange, score =  2: green, score  =  3: blue, score ≥  4: purple. Progression definition for each plot: top row: D5 to D8; bottom row: D9 to D11.

Faster time to sustained progression in patients with lower baseline disability was also observed on several FS (Figure 5). A statistically significant increase in the hazard over all values of the baseline FS score was observed for the pyramidal (*P* = 0.016), sensory (*P* < 0.0001), bowel/bladder (*P* = 0.002), and mental scale (*P* = 0.037). For the cerebellar scale, the time to sustained progression was fastest in patients with a baseline score of 1, and this group was significantly faster than the remaining patients (*P* = 0.037, log-rank test). 

### 3.3. Maintaining Sustained Progression

Since patients who experience sustained progression may not have truly progressed, Table 2 also presents the proportion of patients with sustained progression who maintained the elevated level on the EDSS for the remainder of followup. For definitions based on the EDSS, the more stringent rules were more likely to result in patients maintaining sustained progression, but fewer than 51% of patients maintained sustained progression for each definition. 

Although sustained progression was observed on each FS, the proportion of patients who maintained sustained progression on any FS was lower than the proportion using definitions based on the EDSS. In fact, only 21% of patients who experienced sustained progression on the brainstem FS maintained the elevated level for the remainder of subsequent followup. These results show that definitions of sustained progression based on the FS scales are not sensitive to true irreversible disability. 

When sustained progression on any FS was investigated, the proportion that maintained sustained progression was the highest among any definition. At the same time, the cause of the sustained progression could vary across time points. When the first subscale with sustained progression was required to remain elevated, the proportion that maintained sustained progression was reduced by nearly half (53% versus 28%). The sum of the FS definitions had a similar proportion of people who maintained the elevated level as the definitions based on the EDSS.

In terms of predictors of reversion, the time of subsequent followup was a significant predictor of reversion (*P* < 0.001 for each definition based on the EDSS), but baseline EDSS was not a significant predictor in any model. In particular, patients with longer followup were more likely to revert, demonstrating that our estimated reversion proportions may be underestimated compared to if we had longer followup on all patients. Similar trends were observed for definitions based on the FS. 

### 3.4. Impact of Relapse or Provider Changes on Improvement

Potential explanations for reversions are the presence of relapses or a change in the rating provider. When visits classified during a relapse were removed from the analysis, the results were unchanged. In particular, the proportion of patients who experienced sustained progression was slightly less, but a large percentage of patients still failed to maintain the elevated EDSS or FS scores for the remainder of followup (data not shown). Changing provider was also not significantly associated with reversion since patients who reverted were not significantly more likely to change provider compared to patients who failed to revert (Fisher's exact test  *P* > 0.05 for all tests). Therefore, although a large percentage of our patients who experienced sustained progression failed to maintain progression, relapses and changes in provider were not the main reason for the poor performance of these definitions. 

## 4. Discussion

The analysis of sustained progression in our observational cohort demonstrated that sustained progression by any definition occurs infrequently in the disease modifying therapy era. In addition, sustained progression on the EDSS was only maintained for the remainder of followup for at most 50% of patients, which is consistent with previous findings from the placebo arms of clinical trials [8]. Therefore, the use of any definition presented here as an outcome for clinical trials or observational studies has uncertain clinical meaning in terms of long-term disease progression. A possible reason for the poor performance is the requirement of an elevated EDSS for only two visits to qualify as sustained. Alternative definitions could require elevated EDSS for at least three visits, which corresponds to twelve months in CLIMB patients. All of the previous analyses were also completed requiring progression to be sustained for three consecutive visits, but the results observed were largely unchanged.

One potential explanation for the poor performance of the definitions of progression in this RRMS cohort is the limited relationship between the early phase of the disease and long-term disease progression. A recent study based on the London Ontario cohort demonstrated that only a high relapse rate very early in the disease course was associated with a worse prognosis in terms of progression [13]. A sample of progressive patients from this cohort showed that the rate of progression was roughly the same in patients with no, one, or many relapses in the early phase of the disease [14]. These results demonstrate the lack of association between relapse rate and long-term disease progression. To address the potential problems caused by transient increases in the EDSS due to relapses, the concept of sustained progression was developed, but the inability of any of the definitions presented in this paper to identify true disease progression provides further evidence that short-term changes in the EDSS are likely not identifying true irreversible disability. 

The definitions of sustained progression require the EDSS to remain elevated for consecutive visits to ensure that the increase is not due solely to a relapse. At the same time, the presence of a relapse at the time of sustained progression might explain some of the reversions observed in our dataset. When all observed EDSS measurements taken during a relapse according the scoring provider, the high probability of reversion remained. Therefore, the reversions observed in our sample were not due to relapses and likely show true short-term disease fluctuations.

To address the problem with maintaining sustained progression on the EDSS, sustained progression on each of the FS scales was investigated. Since each FS measures only one system, it was hypothesized that definitions based on a specific FS scale would be less subject to fluctuations. Surprisingly, sustained progression on any individual FS was actually less sensitive to true change than the EDSS. Less than half of patients who experienced sustained progression on a specific FS maintained the progression. Therefore, using the FS scores for future studies does not appear to allow better measurement of irreversible worsening.

The effect of baseline EDSS on the time to sustained progression has important implications for future studies because the probability of sustained progression is a function of the starting EDSS value. Several clinical trials have already incorporated the difference in probability of progression for patients with an EDSS  =  0 through the use of D4 [6, 7], but our results demonstrate that the problem persists for other EDSS values. Therefore, any imbalance in the baseline EDSS may lead to biased estimates of group differences if EDSS is not appropriately controlled in statistical models. The very low chance of sustained progression for patients with an EDSS of 3–3.5 may also be due to regression to the mean/measurement error. Regression to the mean is a well-described phenomenon in MS clinical trials with regard to disease activity [15], but this is rarely considered in analysis of sustained progression. 

Our study has several limitations that warrant further discussion. One limitation is that different neurologists scored patients at different time points over the course of the study. The interrater reliability of the EDSS is lower than the intrarater reliability of the EDSS [16], so some of the observed changes may be due to changes in rater. Although it is impossible to know what the EDSS would have been scored if the same rater was used throughout the study, changing rater was not significantly associated with maintaining sustained progression in this study. This result does not imply that rater had no effect on EDSS score, but it does demonstrate that the reversions observed in our study were not exclusively due to rater changes. A second limitation is that no restrictions were placed on treatment regimens followed by patients. Lastly, our results reflect those of patients enrolled in the CLIMB study. Although patients in the CLIMB study are treated in the same manner as other patients within our clinic, which, to our knowledge is in line with standard practices of tertiary MS Centers in the United States, it is possible that this population may differ from others seen within different practice settings or different geographic locations. 

In conclusion, no definition of sustained progression based on the EDSS or the component FS scores was sensitive for irreversible progression of the disease. Since changing the definition failed to lead to improved performance, alternative measures of disease progression/severity must be found to better classify patients with short-term disease progression.

## Figures and Tables

**Table 1:** Baseline demographic and clinical characteristics in study sample.

*N*	929
Gender (% female)	75
Age (years, mean ± SD)	41 ± 10.5
Disease duration (years, mean ± SD)	7.9 ± 8.1
Race (% white)	95
Follow-up time (months, mean ± SD)	44.7 ± 29.4
EDSS (mean ± SD)	1.22 ± 1.08
Pyramidal scale (mean ± SD)	0.61 ± 0.82
Cerebellar scale (mean ± SD)	0.28 ± 0.66
Brainstem scale (mean ± SD)	0.15 ± 0.43
Sensory scale (mean ± SD)	0.53 ± 0.77
Bowel bladder scale (mean ± SD)	0.27 ± 0.56
Mental scale (mean ± SD)	0.17 ± 0.49
Visual scale (mean ± SD)	0.22 ± 0.66
Treatment (% IFN, % GA, % other, % untreated)	29.7, 30.9, 7.2, 32.2

EDSS: expanded disability status scale, IFN: all forms of interferon-*β*, GA: glatiramer acetate, other: natalizumab, rituximab, cyclophosphamide, daclizumab, mycophenolate mofetil, mitoxantrone, or combination treatment.

**Table 2:** Patients who met each definition of sustained progression during followup.

	Patients with sustained progression		Patient with sustained progression and subsequent visits		Patients who always progressed among those with subsequent visits	
	Number	Percent of all patients	Number	Percent of those with sustained progression	Number	Percent of those with subsequent visits
EDSS						
(D1) Increase of 0.5	392	42.2	315	80.4	137	43.5
(D2) Increase of 1	278	29.9	225	80.9	98	43.6
(D3) Increase of 1.5	147	15.8	120	81.6	61	50.8
(D4) Increase of 1/increase 1.5 for baseline EDSS = 0	212	22.8	169	79.7	73	43.2
Functional system						
(D5) Pyramidal function	217	23.4	179	82.5	53	29.6
(D6) Cerebellar function	120	12.9	94	78.3	29	30.9
(D7) Brainstem function	89	9.6	62	69.7	13	21
(D8) Sensory function	271	29.2	226	83.4	75	33.2
(D9) Bowel bladder function	179	19.3	135	75.4	38	28.1
(D10) Mental function	161	17.3	112	69.6	40	35.7
(D11) Visual function	91	9.8	73	80.2	16	21.9
Combination of functional system						
(D12) Any FS	536	57.7	433	80.8	228	52.7
(D13) First FS	536	57.7	433	80.8	122	28.2
(D14) Sum of subscales increase of 2	243	26.2	187	77	85	45.5
(D15) Sum of subscales increase of 3	148	15.9	111	75	49	44.1

A total of 929 patients contributed to the analysis. EDSS: expanded disability status scale.

**Table 3:** Proportion of patients who experienced sustained increases in functional systems associated with sustained progression on the EDSS.

	(D1) Increase of 0.5 on the EDSS	(D2) Increase of 1 on the EDSS	(D3) Increase of 1.5 on the EDSS	(D4) Increase of 1/1.5 on the EDSS based on baseline EDSS
Total progressed	392	278	147	212
Number (%) progressed on pyramidal scale	119 (30.4)	96 (34.5)	65 (44.2)	88 (41.5)
Number (%) progressed on cerebellar scale	40 (10.2)	27 (9.7)	25 (17.0)	27 (12.7)
Number (%) progressed on brainstem scale	26 (6.6)	21 (7.6)	19 (12.9)	21 (9.9)
Number (%) progressed on sensory scale	140 (35.7)	113 (40.6)	83 (56.5)	104 (49.1)
Number (%) progressed on bowel bladder scale	66 (16.8)	55 (19.8)	39 (26.5)	55 (25.9)
Number (%) progressed on mental scale	59 (15.1)	46 (16.5)	31 (21.1)	44 (20.8)
Number (%) progressed on visual scale	32 (8.2)	26 (9.4)	20 (13.6)	27 (12.7)
Number (%) progressed on multiple scales	124 (31.6)	105 (37.8)	92 (62.6)	118 (55.7)
Number (%) progressed on no scale	81 (20.7)	47 (16.9)	5 (3.4)	15 (7.1)

EDSS: expanded disability status scale.
